# Anti-*Helicobacter*, Antitubercular and Cytotoxic Activities of Scalaranes from the Red Sea Sponge *Hyrtios erectus* †

**DOI:** 10.3390/molecules23040978

**Published:** 2018-04-23

**Authors:** Abdulrahman M. Alahdal, Hani Z. Asfour, Safwat A. Ahmed, Ahmad O. Noor, Ahmed M. Al-Abd, Mahmoud A. Elfaky, Sameh S. Elhady

**Affiliations:** 1 Department of Clinical Pharmacy, Faculty of Pharmacy, King Abdulaziz University, Jeddah 21589, Saudi Arabia; aalahdal2@hotmail.com (A.M.A.); aonoor@kau.edu.sa (A.O.N.); 2 Department of Medical Microbiology and Parasitology, Faculty of Medicine, Princess Al-Jawhara Center of Excellence in Research of Hereditary Disorders, King Abdulaziz University, Jeddah 21589, Saudi Arabia; hasfour@kau.edu.sa; 3 Department of Pharmacognosy, Faculty of Pharmacy, Suez Canal University, Ismailia 41522, Egypt; safwat_aa@yahoo.com; 4 Department of Pharmacology and Toxicology, Faculty of Pharmacy, King Abdulaziz University, Jeddah 21589, Saudi Arabia; ahmedmalabd@pharma.asu.edu.eg; 5 Department of Natural Products and Alternative Medicine, Faculty of Pharmacy, King Abdulaziz University, Jeddah 21589, Saudi Arabia; melfaky@kau.edu.sa; 6 Department of Pharmacognosy, Faculty of Pharmacy, Port Said University, Port Said 42526, Egypt

**Keywords:** sponges (Porifera), *Hyrtios erectus*, scalarane sesterterpenoids, 12-*O*-deacetyl-12,19-di-*epi*-scalarin, *Helicobacter pylori*, antitubercular, cytotoxic

## Abstract

The Red Sea specimen of the marine sponge *Hyrtios erectus* (order Dictyoceratida) was found to contain scalarane-type sesterterpenes. 12-*O*-deacetyl-12,19-di-*epi*-scalarin (**14**), a new scalarane sesterterpenoid, along with fourteen previously-reported scalarane-type sesterterpenes (**1**–**13** and **15**) have been isolated. The chemical structures of the isolated compounds were elucidated on the basis of detailed 1D and 2D NMR spectral data and mass spectroscopy, as well as by comparison with reported data. The anti-*Helicobacter pylori*, antitubercular and cytotoxic activities of all fifteen compounds were evaluated to reveal the potency of Compounds **1**, **2**, **3**, **4**, **6**, **7** and **10**. Amongst these, Compounds **1**, **3**, **4**, **6** and **10** displayed a promising bioactivity profile, possessing potent activities in the antitubercular and anti-*H. pylori* bioassay. Compounds **2** and **7** showed the most promising cytotoxic profile, while Compounds **1** and **10** showed a moderate cytotoxic profile against MCF-7, HCT-116 and HepG2 cell lines.

## 1. Introduction

Marine sponges of the genus *Hyrtios*, order Dictyoceratida, family Thorectidae, occur often in tropical and subtropical sea waters and frequently form the dominant habitat on coral reefs. Previous biological and chemical investigations of the genus *Hyrtios* and their derived microbial symbionts have showed the presence of numerous natural products possessing a variety of important biological activities and unique structure including scalarane-type sesterterpenes [1,2,3,4,5,6,7,8,9,10,11,12,13,14,15,16,17,18,19,20,21], sesquiterpenes [22,23,24], acyclic triterpenoids [25], macrolides [26,27,28,29], steroids [30], indole and β-carboline alkaloids [24,31,32,33,34,35,36,37,38]. Many sesterterpenoids of the scalarane class showed a variety of pharmacological activities. Among these is the sesterterpene heteronemin [14,39], which exhibited antimycobacterial activity against *M. tuberculosis* H_37_Rv with a MIC value of 6.25 µg/mL [40]. Other biological activities of scalaranes include cytotoxic [3,4,5,13,18,19,20,41,42], antifeedant [43,44], antitubercular [6], antimicrobial [45,46], ichthyotoxic [47], anti-inflammatory [14,48], platelet aggregation inhibition [49,50] and nerve growth factor synthesis-stimulation [21,51].

**Figure 1:**
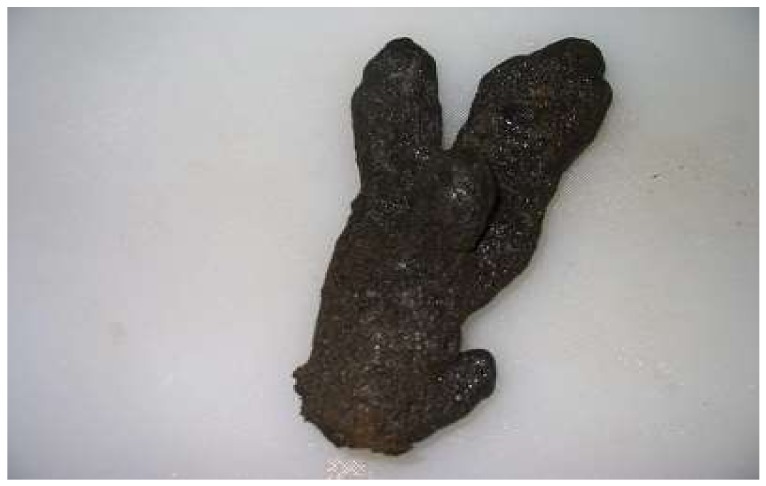
Red Sea sponge *Hyrtios erectus* (morphology before methanol extraction).

In the course of our ongoing efforts to identify drugs from the sea [2,3,52], we have studied and investigated the extract of the Red Sea marine sponge *Hyrtios erectus* (Figure 1). We report herein the characterization, anti-*H*. *Pylori*, antitubercular and cytotoxic activities of fifteen scalarane-type sesterterpenes including the new compound 12-*O*-deacetyl-12,19-di-*epi*-scalarin (**14**), together with the previously-reported compounds, sesterstatin 7 (**1**) [6], heteronemin (**2**) [39], scalarolide (**3**) [43], 12-*epi*-24-deoxyscalarin (**4**) [53], scalarolide acetate (**5**) [46], 19 acetylsesterstatin 3 (**6**) [4], 12-deacetyl-12,18-di-*epi*-scalaradial (**7**) [43], 12-deacetyl-12-*epi*-scalaradial (**8**) [14], sesterstatin 3 (**9**) [20], 12*β*,20*α*-dihydroxy-16*β*-acetoxy-17-scalaren-19,20-olide (**10**) [54], 12-*O*-acetyl-16-*O*-methylhyrtiolide (**11**) [55], 12*β*-acetoxy,16*β*-methoxy,20*α*-hydroxy-17-scalaren-19,20-olide (**12**) [3], 24-methoxypetrosaspongia C (**13**) [2] and 12-acetoxy,16-*epi*-hyrtiolide (**15**) [3]. The previously-known compounds exhibited diverse cytotoxic activity against several cancer cell lines.

## 2. Results and Discussion

### 2.1. Purification of Compounds **1**–**15**

Successive fractionation of the lipophilic fraction obtained from the methanolic extract of the sponge using silica gel column chromatography followed by final purification on semipreparative reversed phase HPLC column afforded fifteen pure isolated compounds (**1**–**15**) containing a scalarane-type framework, of which Compound **14** was assigned as a new scalarane sesterterpenoid.

### 2.2. Structural Elucidation of Compounds **1**–**15**

**Figure 2:**
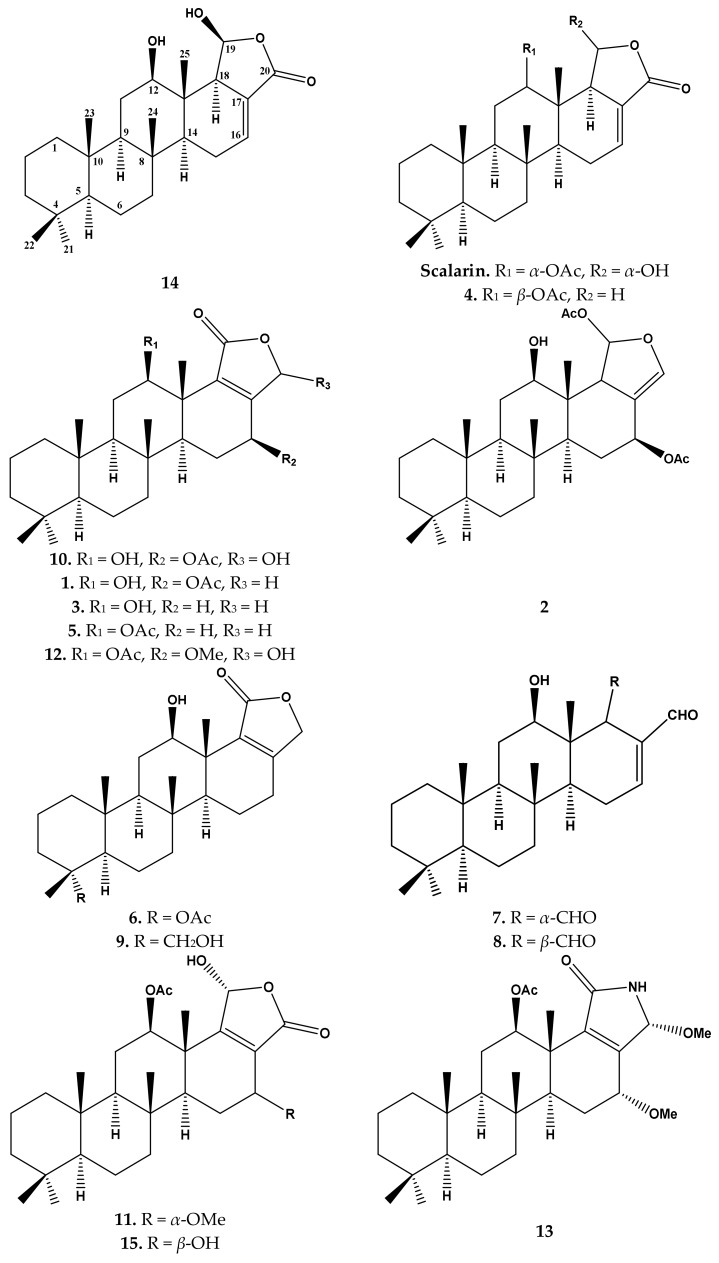
Scalarane Sesterterpenes **1**–**15**.

**Figure 3:**
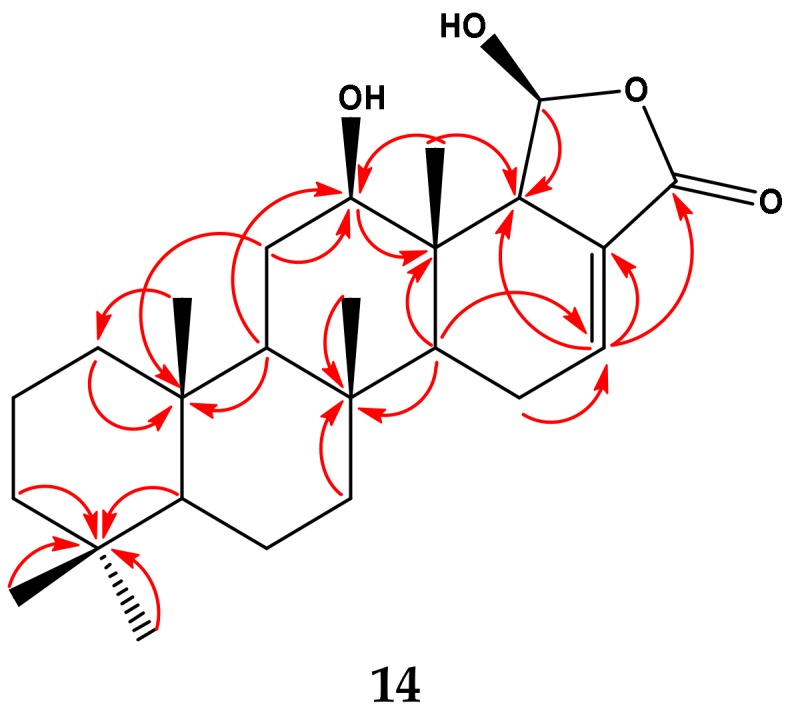
Selected HMBC correlations observed for Compound **14**.

Compound **14** (Figure 2) was isolated and purified as an amorphous powder. The molecular formula, C_25_H_38_O_4_, was established from the positive ESIMS, as well as from ^13^C-NMR data. In the mass spectrum of Compound **14**, the molecular ion peak was of low abundance or absent, but strong peaks corresponding to losses of water were observed at *m*/*z* 385.3 [C_25_H_36_O_3_ (M − H_2_O)⁺] and *m*/*z* 367.3 [C_25_H_35_O_2_ (M + H − 2H_2_O)⁺] (Supplementary Materials, Figure S1). The ^1^H and ^13^C-NMR spectra measured in CDCl_3_ (Table 1), as well as correlations in the HMBC (Table 1, Figure 3) suggested that Compound **14** was also a scalarane-type sesterterpenoid. Thus, five singlets at *δ*_H_ 0.81, 0.83, 0.85, 0.86 and 0.93 were assigned to the five methyl groups of a scalarane skeleton. In the ^13^C-NMR spectrum of **14** (Table 1), the carbon signals in the low-field region at *δ*_C_ 98.5 (C-19), 127.3 (C-17), 136.3 (C-16) and 166.9 (C-20) were reminiscent of those of scalarin [56] (Figure 2).

The NMR spectra of Compound **14** (Supplementary Materials, Figures S2–S5) were quite similar to those of scalarin [56] (Figure 2). However, several diagnostic differences were observed. The most evident were the absence of the signals due to the acetyl group, the upfield shift of H-12 at *δ*_H_ 3.57, the chemical shift of CH-18 at *δ*_C_ 58.7, *δ*_H_ 2.54/(*δ*_C_ 50.8, *δ*_H_ 3.14, in scalarin), and the ^13^C chemical shift of CH-19 at *δ*_C_ 98.5/(*δ*_C_ 98.9 in scalarin).

**Figure 4:**
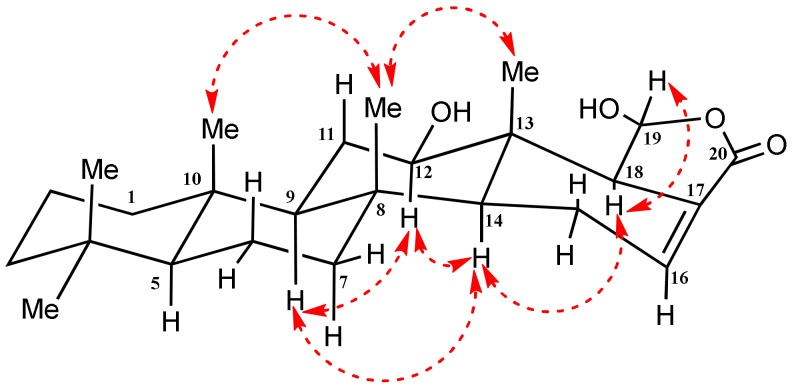
Important NOESY NMR correlations observed for Compound **14**.

The relative configuration of H-12 and H-19 was detected by coupling constants (for H-12) and confirmed by interpreting the NOESY spectrum (Supplementary Materials, Figure S6). The α-configuration of H-12 was deduced on the basis of the diaxial coupling of H-12 (δ_H_ 3.57; dd, *J* = 11.05 and 4.25 Hz) with H-11 and cross-peaks with α-oriented H-9 and H-14 in NOESY (Figure 4). Finally, the signal assigned to H-19 was correlated in the NOESY spectrum with H-18, which in turn was correlated with H-14, and H-12 indicated the α orientation of H-19 (Figure 4); therefore, the configuration at the C-12/C-19 carbons was opposite to that of scalarin (Figure 2). To the best of our knowledge, Compound **14** has not been reported before in the literature; therefore, it is considered as a new scalarane sesterterpene analogue. The name 12-*O*-deacetyl-12, 19-di-*epi*-scalarin was assigned for this compound.

The other known Compounds **1**–**13** and **15** (Figure 2) were identified by extensive study of their spectral data, including ESIMS, 1D and 2D NMR data, as well as by comparison with the published data.

### 2.3. Biological Activities of the Isolated Compounds **1**–**15**

The isolated Compounds **1**–**15** were evaluated for their antitubercular, anti-*H*. *pylori* and cytotoxic activities (Table 2). Anti-*H*. *pylori* compounds are reported herein for the first time for scalarane sesterterpene analogues. Among these, Compounds **1**, **3**, **4**, **6** and **10** displayed a promising bioactivity profile, possessing potent activities in the antitubercular and anti-*H. pylori* bioassays. Compound **7** displayed an interesting bioactivity profile, possessing potent antitubercular and cytotoxic activities and being practically slightly active in the anti-*H. pylori* bioassay.

In the cytotoxic assay, the compounds tested against various cell lines (MCF-7, HCT-116 and HepG2) showed variable cytotoxic activity. Amongst these, Compounds **2** and **7** showed the most promising cytotoxic profile with IC_50_ values ranging from 0.4 ± 0.1–1.2 ± 0.1 µM and from 1.4 ± 0.05–1.6 ± 0.1 µM against the cell lines under investigation, respectively. Compounds **1**, **5** and **10** showed a moderate cytotoxic profile with IC_50_ values less than, or approximately, 20 µM against all cell lines under investigation. Other compounds showed weak to no activity against the cell lines under investigation (Table 2).

## 3. Materials and Methods

### 3.1. General Experimental Procedures

Optical rotation was measured on the automatic high-speed laboratory polarimeter P3000 (A.KRUSS Optronic Gmbh, Hamburg, Germany). UV spectra were measured on a Hitachi 300 Spectrophotometer (Hitachi High-Technologies Corporation, Kyoto, Japan). High-resolution ESIMS data were recorded with an ultra-high resolution (UHR) TOF spectrometer (Impact, Bruker, Bremen, Germany). NMR spectra were obtained in CDCl_3_ on a Bruker Avance DRX 600-MHz spectrometer (Bruker, Bremen, Germany) at 600 MHz for ^1^H-NMR and 150 MHz for ^13^C-NMR. NMR chemical shifts were expressed in parts per million (ppm) referenced to residual CDCl_3_ solvent signals (*δ*_H_ 7.26 for ^1^H and *δ*_C_ 77.0 for ^13^C). Precoated SiO_2_ 60 F_254_ plates (Merck, Darmstadt, Germany) were used for TLC. For column chromatography, SiO_2_ (70–230 mesh, Merck, Darmstadt, Germany) was used. HPLC purifications were performed on an HPLC column (5-µm ZORBAX Eclipse XDB-C18, 250 × 4.6 mm, Agilent, Santa Clara, CA, USA).

### 3.2. Biological Materials

The marine sponge specimens used in this study (Figure 1) were collected from the Red Sea, Egypt, by scuba diving. The sponge material was immediately frozen after collection and kept at −20 °C until investigation. The sponge specimen was later identified to be *Hyrtios erectus* (class: Demospongiae, order: Dictyoceratida, family: Thorectidae) by Dr. Rob van Soest (Institute of Systematic Population Biology, Amsterdam University, The Netherlands). A voucher specimen was preserved at the Zoological Museum of the University of Amsterdam, under Registration Number ZMAPOR19761.

### 3.3. Purification of Compounds **1**–**15**

The collected sponge specimen (0.90 kg, wet wt.) was cut into small pieces and was macerated exhaustively at room temperature in MeOH. The combined extracts were concentrated under reduced pressure to yield the organic crude extract (85 g). The total crude extract was subjected to silica gel column using VLC (vacuum liquid chromatography) gradient elution (*n*-hexane–CHCl_3_–MeOH) to afford Fractions 1–9.

Fraction 4 (*n*-hexane–CHCl_3_, 1:3) was chromatographed on silica gel column with *n*-hexane–CHCl_3_–MeOH gradient elution to give 8 further fractions. Of these, Fraction 3 (700 mg) was fractionated over a silica gel column chromatography (*n*-hexane–CHCl_3_), then finally purified by HPLC (ODS XDP-Zorbax column, 5 µm, 250 × 4.6 mm, 80% CH_3_CN/H_2_O, 1.5-mL/min flow rate and 220-nm UV detection), and Compounds **1**–**8** were obtained (4.6, 14, 3.4, 3, 4, 2.9, 2.3 and 3.5 mg, respectively). In turn, Fraction 5 (137 mg) was further chromatographed on silica gel column chromatography followed by final purification on HPLC the same as Fraction 3, and compounds **9**–**14** were obtained (2.2, 4, 3, 2.3, 2.7 and 1.3 mg, respectively). Finally, Fraction 6 (55 mg) was also chromatographed and purified as Fraction 3 to obtain Compound **15** (3.5 mg).

Compound **14**: Amorphous solid (1.3 mg); $\alpha_{D}^{25}$ +112.0 (*c* 0.1, CHCl_3_); UV (λ_max_, MeOH) (log ε): 226 (4.31), 285 (2.54) nm; NMR data: see Table 1; ESI-MS: *m*/*z* 385.3 [M − H_2_O]⁺. HRESIMS: *m*/*z* 403.2851 (calculated for C_25_H_39_O_4_ [M + H]⁺, 403.2848).

### 3.4. Biological Activity of Compounds **1**–**15**

#### 3.4.1. Anti-*Helicobacter pylori* Activity Assessment

The anti-*H*. *pylori* activity was assessed against a strain of *Helicobacter pylori* (American Type Culture Collection, H.b., ATCC 700392) using a micro-well dilution method as previously described [57]. Clarithromycin was used as a positive control and exhibited an MIC of 1.31 µM.

#### 3.4.2. Antitubercular Activity Assessment

A nonvirulent strain *Mycobacterium tuberculosis* (ATCC 25177, H37Ra) was obtained from the American Type Culture Collection. The antitubercular activity was assessed according to the protocol described by Franzblau [58,59] using the microplate Alamar blue assay (MABA). Isoniazid was used as positive control and exhibited an MIC of 0.87 µM.

#### 3.4.3. Cytotoxic Activity Assessment

##### 3.4.3.1. Cell Culture

Human hepatocellular carcinoma cells (HepG2), colorectal adenocarcinoma cells (HCT-116) and human breast adenocarcinoma cells (MCF-7) were obtained from the VACSERA (Giza, Egypt). HCT-116 cells were maintained in Roswell Park Memorial Institute Media (RPMI-1640), while HepG2 and MCF-7 cells were maintained in Dulbecco Modified Eagle’s Media (DMEM). All culture media were supplemented with heat-inactivated fetal bovine serum (10% *v/v*), penicillin-G (100 units/mL) and streptomycin sulfate (100 µg/mL). Cells were passaged in a humidified chamber (37 °C and 5% (*v/v*) CO_2_).

##### 3.4.3.2. Trypan-Blue Exclusion Assay

Cell viability was measured prior to seeding using the trypan-blue exclusion method. Briefly, exponentially-growing cells were trypsinized by 0.25% trypsin-EDTA solution. Aliquots of cell suspensions were mixed with trypan blue solution (0.4% *w*/*v*), and blue color (positive cells) was determined. Cells were not seeded unless viability was greater than 95%.

##### 3.4.3.3. Cytotoxic Assessment

The cytotoxic effects of Compounds **1**–**15** on breast (MCF-7), colorectal (HCT-116) and liver cancer cells (HepG2) were evaluated using the sulforhodamine B (SRB) method as previously described [60]. Briefly, mid-exponentially-proliferating cells were trypsinized by trypsin-EDTA (0.25% *w*/*v*) and seeded in 96-well plates (1000–2000 cells/well). Cells were treated with serial concentrations of the compounds under investigation for 72 h and subsequently fixed with TCA (10% *w*/*v*) for 1 h at 4 °C. After washing three times, cells were stained with 0.4% SRB solution for 10 min in a dark place and then washed with glacial acetic acid (1% *v*/*v*). After drying, Tris-HCl (50 mM, pH 7.4) was used to dissolve the SRB-stained cells, and color intensity was measured at 540 nm. The dose response relationship of test compounds was analyzed using the *E*_max_ model (Equation (1)).

(1) $$\%\;\mathrm{cell}\;\mathrm{viability}=\left(100-R\right)\times \left(1-\frac{{\left[D\right]}^{m}}{{K_{d}}^{m}+{\left[D\right]}^{m}}\right)+R$$

where (*R*) is the residual unkilled fraction (the resistance fraction), (*D*) is the drug concentration used, (*K_d_*) or IC_50_ is the drug concentration needed to produce 50% viability reduction and (m) is the Hill-type coefficient. IC_50_ is defined as the drug concentration required to reduce maximum absorbance by 50% compared to untreated control cells [61].

#### 3.4.3.4. Statistical Analysis

Data are presented as the mean ± SEM using GraphPad prism™ software Version 5.00 (GraphPad software Inc., La Jolla, CA, USA) for Windows Version 10.00. Analysis of variance (ANOVA) with the Tukey–Kremer post hoc test was used to calculate the significance using SPSS^®^ for Windows, Version 17.0.0. *p* < 0.05 was taken as a cut-off value for significance.

## 4. Conclusions

Chemical investigation of the bioactive extract of the sponge *Hyrtios erectus*, collected in the Red Sea, Egypt, yielded fifteen compounds of scalarane-type sesterterpenes. Compounds **1**–**15** including a new one (**14**) were purified, and their chemical structures were characterized using spectroscopic studies. The isolated compounds belong to the class of scalarane sesterterpenes and displayed diverse biological activities including anti-*H*. *pylori*, antitubercular and cytotoxic. Anti-*H*. *pylori* compounds are reported herein for the first time for scalarane sesterterpene analogues. Among these, Compounds **1**, **3**, **4**, **6** and **10** displayed a promising bioactivity profile, possessing potent activities in the antitubercular and anti-*H. pylori* bioassays. Compound **7** displayed an interesting bioactivity profile, possessing potent antitubercular and cytotoxic activities and being practically slightly active in the anti-*H. pylori* bioassay. Moreover, Compounds **2** and **7** showed the most promising cytotoxic profile, while Compounds **1**, **5** and **10** showed a moderate cytotoxic profile against MCF-7, HCT-116 and HepG2 cell lines. Other compounds showed weak to no activity against the cell lines under investigation.

## Figures and Tables

**Table 1:** NMR data and HMBC correlations of Compound **14** (CDCl_3_).

Position	*δ*_C_	*δ*_H_ (m, *J* in Hz)	HMBC (H→C) ^a^
1	39.8, CH_2_	1.73, 0.83 (m)	C-10
2	18.5, CH_2_	1.63, 1.46 (m)	C-4, C-10
3	42.0, CH_2_	1.39, 1.15 (m)	C-4
4	33.2, C	-	-
5	56.4, CH	0.83 (m)	C-4
6	18.0, CH_2_	1.58, 1.42 (m)	
7	41.4, CH_2_	1.74, 0.95 (m)	C-8
8	37.4, C	-	-
9	58.8, CH	0.93 (m)	C-10, C-12
10	37.4, C	-	-
11	26.1, CH_2_	1.79, 1.50 (m)	C-10, C-12
12	80.4, CH	3.57 (dd, 11.05, 4.25)	C-9, C-11, C-18, C-25
12-O*H*		4.19 (s)	
13	39.9, C	-	-
14	52.7, CH	1.25 (m)	C-8, C-9, C-13, C-16, C-18
15	23.5, CH_2_	2.17, 2.37 (m)	C-16
16	136.3, CH	6.87 (dd, 6.80, 3.40)	C-14, C-15, C-18, C-20
17	127.3, C	-	-
18	58.7, CH	2.54 (m)	C-12, C-13, C-25
19	98.5, CH	5.74 (d, 5.10)	C-18
20	166.9, C	-	-
21	21.3, CH_3_	0.81 (s)	C-4
22	33.2, CH_3_	0.83 (s)	C-4
23	16.5, CH_3_	0.85 (s)	C-1, C-5, C-9, C-10
24	16.7, CH_3_	0.93 (s)	C-7, C-8, C-9, C-14
25	9.1, CH_3_	0.86 (s)	C-12, C-13, C-14, C-18

^a^ HMBC correlations are from proton(s) stated for the indicated carbons.

**Table 2:** Anti-*H. pylori*, antitubercular and cytotoxic activities of Compounds **1**–**15** in µM.

Compound	Anti-*H*. *pylori* (MIC)	Anti-TB (MIC)	Cytotoxic (IC_50_ ± SEM)		
			MCF-7	HCT-116	HepG2
**1**	4.39	0.54	24.6 ± 2.3	25.5 ± 3.3	19.8 ± 1.4
**2**	NA	16.00	1.2 ± 0.1	0.4 ± 0.1	1.1 ± 0.1
**3**	10.10	5.05	NA	NA	NA
**4**	9.11	1.12	25.9 ± 1.9	17.5 ± 1.3	24.7 ± 4.8
**5**	146.02	9.13	22.0 ± 0.4	15.2 ± 2.0	15.3 ± 1.1
**6**	8.78	4.39	-	-	-
**7**	20.23	5.05	1.6 ± 0.1	1.4 ± 0.05	1.6 ± 0.5
**8**	80.95	10.12	32.7 ± 3.2	34.5 ± 7.5	23.5 ± 1.8
**9**	77.73	19.42	NA	NA	NA
**10**	8.47	4.23	12.4 ± 0.9	2.9 ± 0.7	5.5 ± 0.9
**11**	263.71	16.47	55.6 ± 2.1	17.8 ± 1.9	20.1 ± 1.5
**12**	32.97	8.24	37.3 ± 5.5	22.8 ± 2.2	34.9 ± 6.1
**13**	16.03	8.02	54.2 ± 3.3	26.5 ± 1.1	26.6 ± 3.8
**14**	81.38	20.33	NA	NA	NA
**15**	33.97	16.97	34.9 ± 4.9	48.6 ± 7.2	27.3 ± 3.3
**isoniazid**	----	0.87	----	----	----
**clarithromycin**	1.31	----	----	----	----
**doxorubicin**	----	----	0.41 ± 0.1	0.11 ± 0.04	0.85 ± 0.1

NA: No activity within the range of concentration used; data are presented as the mean ± SD; *n* = 3.

## Supplementary Materials

The supplementary materials are available online.
