# Supplementary material for: Anti-Helicobacter, Antitubercular and Cytotoxic Activities of Scalaranes from the Red Sea Sponge Hyrtios erectus
Source: Molecules. 2018 Apr 23;23(4):978. doi: 10.3390/molecules23040978 (PMC6017761; doi:10.3390/molecules23040978)
Supplement: Supplementary file 1 [file molecules-23-00978-s001.pdf]

Anti-*Helicobacter*, Antitubercular and Cytotoxic Activities of Scalaranes from the Red Sea Sponge *Hyrtios erectus*

Abdulrahman M. Alahdal [1], Hani Z. Asfour [2], Safwat A. Ahmed [3], Ahmed O. Noor [1], Ahmed M. Al-Abd [4], Mahmoud A. Elfaky [5] and Sameh S. Elhady [5,*]

Additional Experimental Detail.

1) **Figure S1**. ESIMS spectrum of compound **14**
2) **Figure S2**. ^{1}H-NMR spectrum of compound **14** ($CDCl_3$)
3) **Figure S3**. ^{13}C-NMR spectrum of compound **14** ($CDCl_3$)
4) **Figure S4**. HSQC spectrum of compound **14** ($CDCl_3$)
5) **Figure S5**. HMBC spectrum of compound **14** ($CDCl_3$)
6) **Figure S6**. NOESY spectrum of compound **14** ($CDCl_3$)

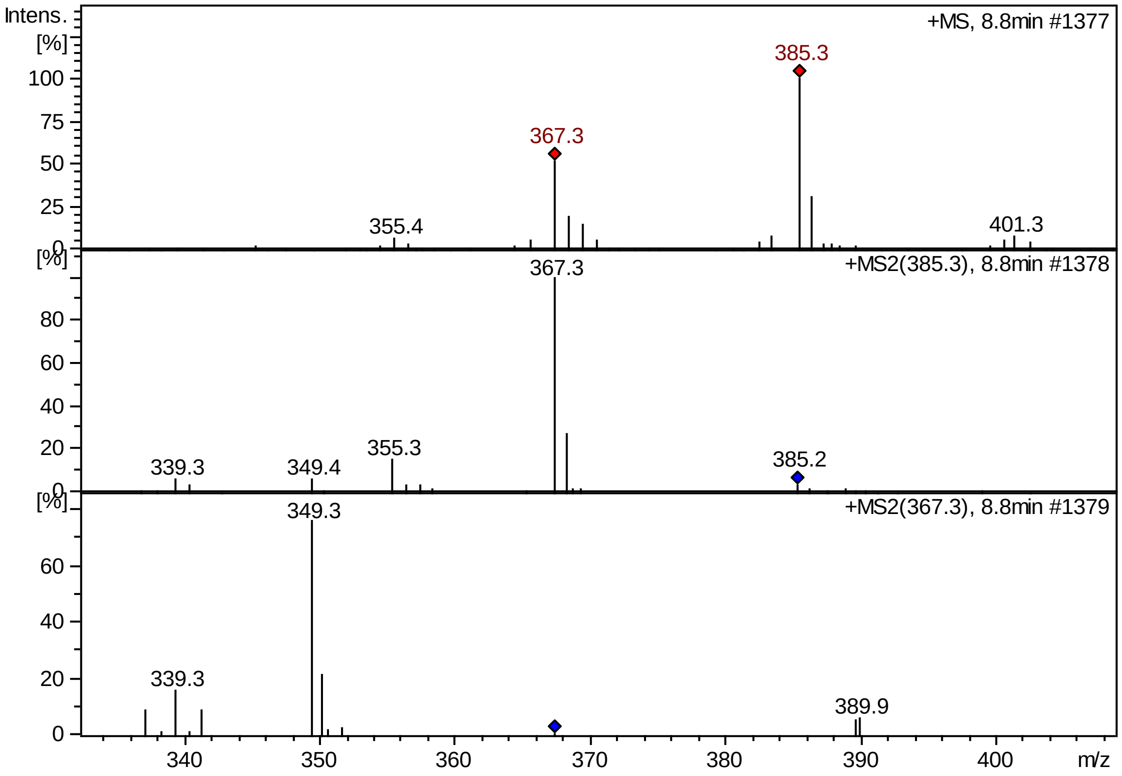

Figure S1. ESIMS spectrum of compound **14**

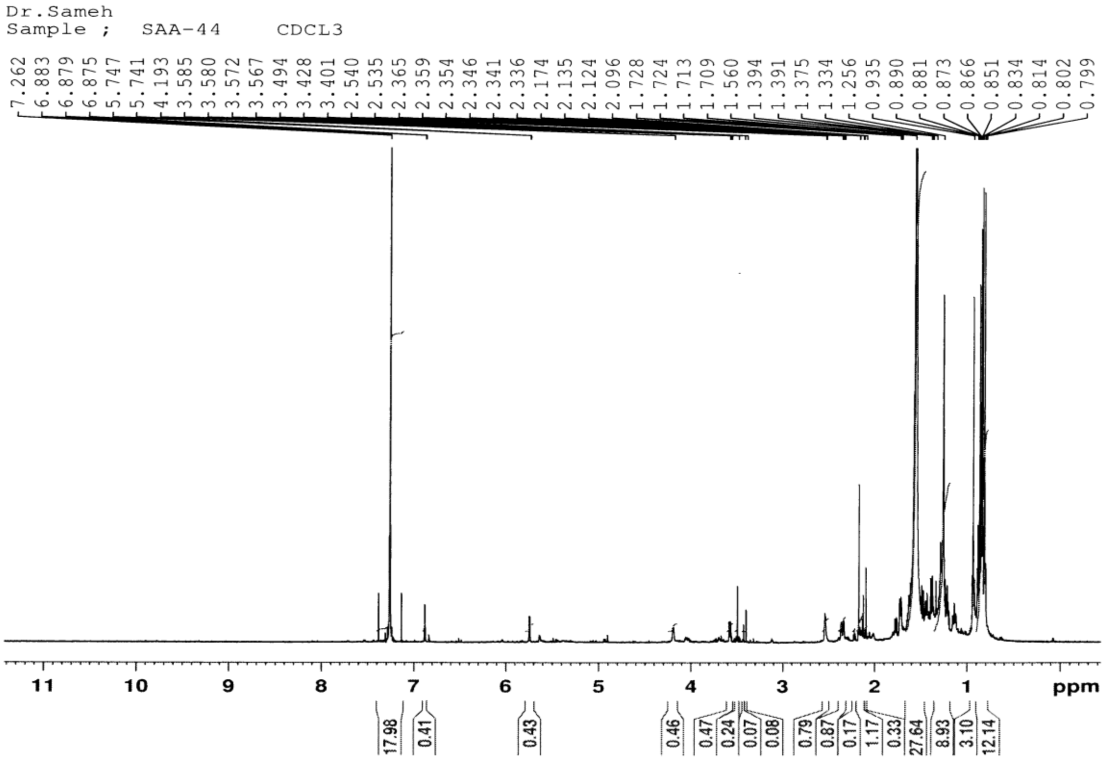

Figure S2. ^{1}H-NMR spectrum of compound **14** ($CDCl_3$)

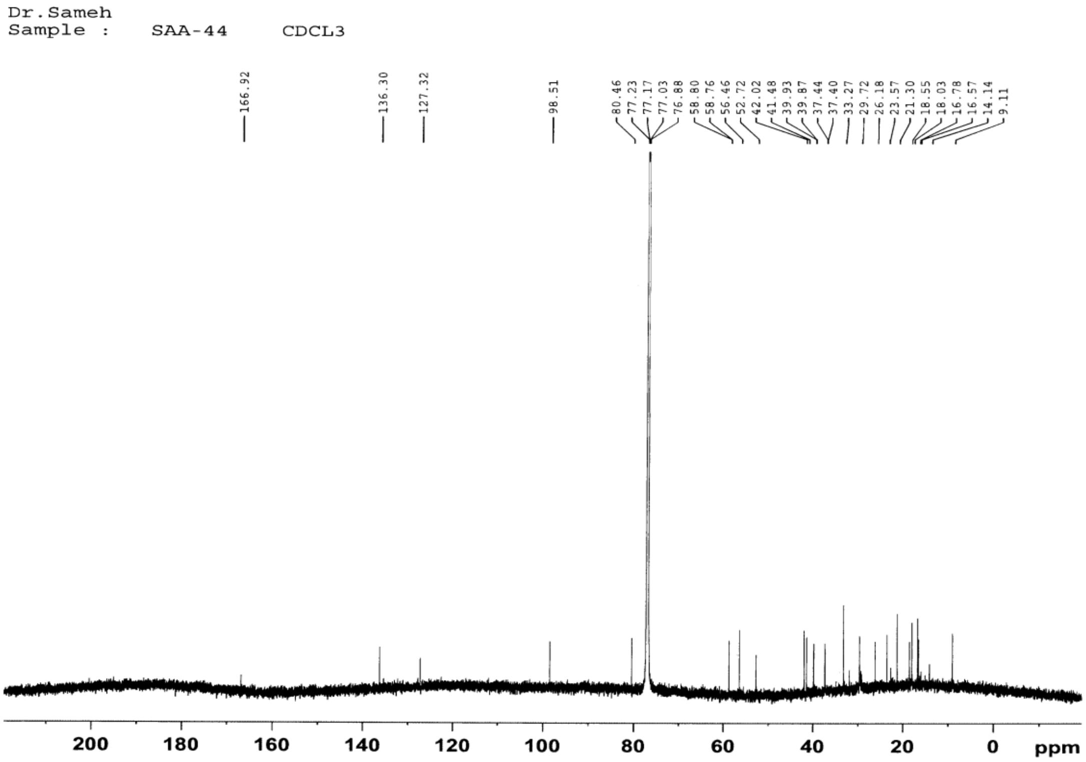

Figure S3. ^{13}C-NMR spectrum of compound **14** ($CDCl_3$)

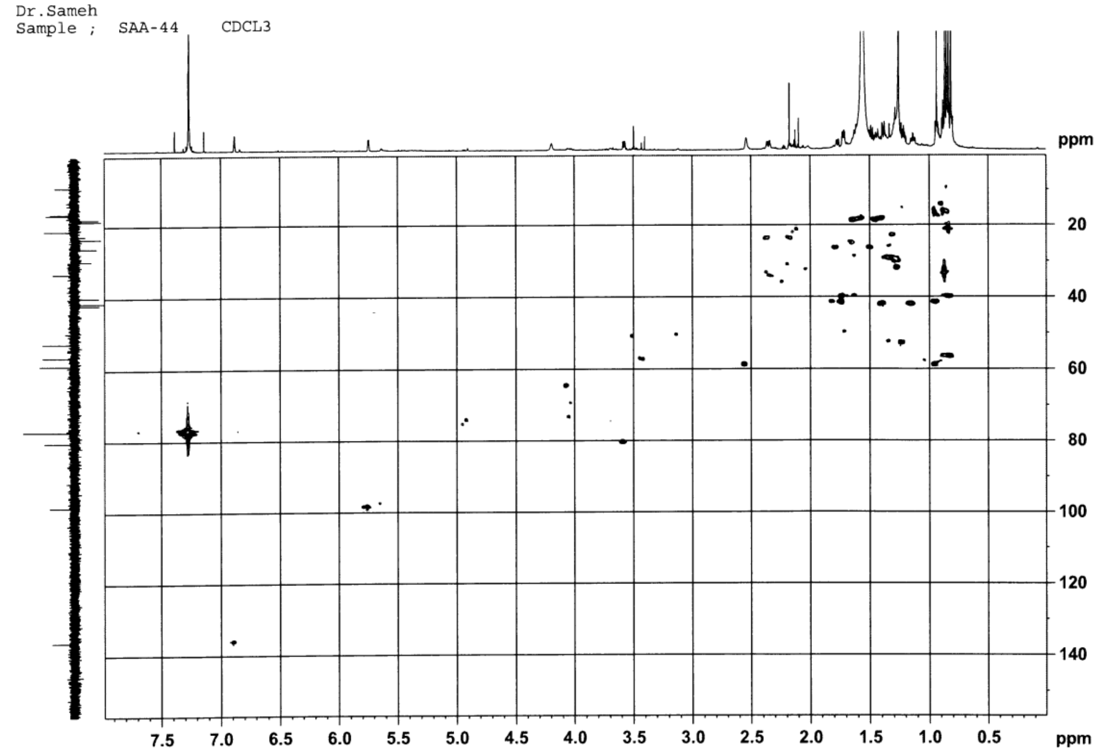

Figure S4. HSQC spectrum of compound **14** ($CDCl_3$)

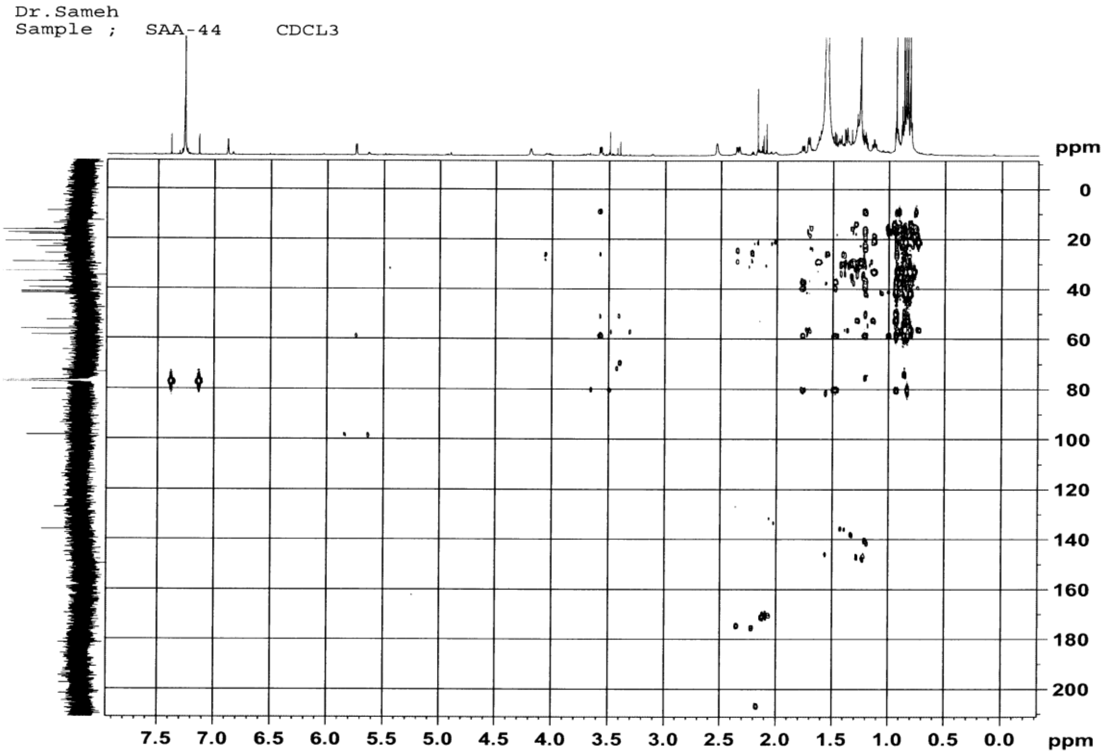

Figure S5. HMBC spectrum of compound **14** ($CDCl_3$)

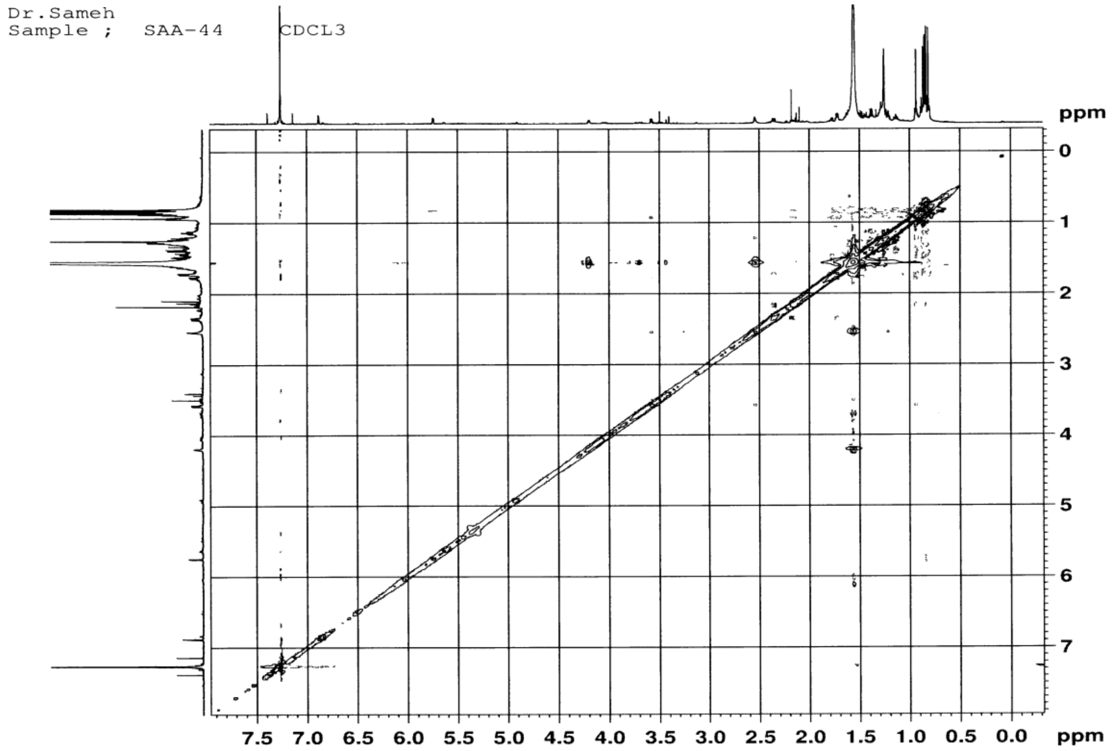

Figure S6. NOESY spectrum of compound **14** ($CDCl_3$)
